# Mammographic Breast Density and Common Genetic Variants in Breast Cancer Risk Prediction

**DOI:** 10.1371/journal.pone.0136650

**Published:** 2015-09-24

**Authors:** Charmaine Pei Ling Lee, Hyungwon Choi, Khee Chee Soo, Min-Han Tan, Wen Yee Chay, Kee Seng Chia, Jenny Liu, Jingmei Li, Mikael Hartman

**Affiliations:** 1 NUS Graduate School for Integrative Sciences and Engineering, National University of Singapore, Singapore, Singapore; 2 Saw Swee Hock School of Public Health, National University of Singapore, Singapore, Singapore; 3 National Cancer Centre, Singapore, Singapore; 4 Human Genetics, Genome Institute of Singapore, Singapore, Singapore; 5 Department of Surgery, National University Hospital, Singapore, Singapore; 6 Department of Medical Epidemiology and Biostatistics, Karolinska Institutet, Stockholm, Sweden; Indiana University, UNITED STATES

## Abstract

**Introduction:**

Known prediction models for breast cancer can potentially by improved by the addition of mammographic density and common genetic variants identified in genome-wide associations studies known to be associated with risk of the disease. We evaluated the benefit of including mammographic density and the cumulative effect of genetic variants in breast cancer risk prediction among women in a Singapore population.

**Methods:**

We estimated the risk of breast cancer using a prospective cohort of 24,161 women aged 50 to 64 from Singapore with available mammograms and known risk factors for breast cancer who were recruited between 1994 and 1997. We measured mammographic density using the medio-lateral oblique views of both breasts. Each woman’s genotype for 75 SNPs was simulated based on the genotype frequency obtained from the Breast Cancer Association Consortium data and the cumulative effect was summarized by a genetic risk score (GRS). Any improvement in the performance of our proposed prediction model versus one containing only variables from the Gail model was assessed by changes in receiver-operating characteristic and predictive values.

**Results:**

During 17 years of follow-up, 680 breast cancer cases were diagnosed. The multivariate-adjusted hazard ratios (95% confidence intervals) were 1.60 (1.22–2.10), 2.20 (1.65–2.92), 2.33 (1.71–3.20), 2.12 (1.43–3.14), and 3.27 (2.24–4.76) for the corresponding mammographic density categories: 11-20cm^2^, 21-30cm^2^, 31-40cm^2^, 41-50cm^2^, 51-60cm^2^, and 1.10 (1.03–1.16) for GRS. At the predicted absolute 10-year risk thresholds of 2.5% and 3.0%, a model with mammographic density and GRS could correctly identify 0.9% and 0.5% more women who would develop the disease compared to a model using only the Gail variables, respectively.

**Conclusion:**

Mammographic density and common genetic variants can improve the discriminatory power of an established breast cancer risk prediction model among females in Singapore.

## Introduction

The primary goal of breast cancer screening is to enable early detection of disease so that prognosis can be improved by more timely intervention. Although screening offers the benefit of reduced mortality for potential patients, the level of over-diagnosis and subsequent treatment of healthy individuals is worthy of concern: for every breast cancer death prevented, three women would be unnecessarily treated for the disease [1]. More importantly, a large majority of women who go for screening are never diagnosed with breast cancer. This suggests a need for re-allocation of resources so that women at higher risk of developing breast cancer are accorded more frequent supervision as a preventive measure.

To facilitate the decision-making process, a woman’s risk is often assessed using her demographic and reproductive information, as well as the presence of a positive family history [2]. One of the most established prediction models used is the Gail model, which considers a woman’s family history, age, age at menarche, age at first live birth, and number of breast biopsies to provide an individualized estimate of breast cancer risk [3]. Subsequently, the model has been modified for use in other ethnic populations [4–9]. Although these models were well-calibrated, their discriminatory power was relatively poor with areas under the curve (AUCs) of the receiver-operating characteristic (ROC) ranging from 0.53 to 0.66 [2,10].

In a recent study, Chay *et al* evaluated the applicability of the Gail model in Singapore. They found that the Gail model over-estimated the population’s 10-year breast cancer risk by 85% overall, with women aged 60–64 having a predicted incidence that was thrice as high as observed [11]. This report highlights the need for an alternative model that is generalizable to Asian communities where the uptake of mammography screening and incidence of breast cancer is substantially lower [12,13].

Apart from the established reproductive factors, other variables have also been reported to be associated with breast cancer. Mammographic density has consistently been shown to be an independent and strong modifiable risk factor, increasing one’s risk of breast cancer by 3 to 6 times [14–20] for at least a decade [18]. Several groups have evaluated the impact of including mammographic density in breast cancer risk assessment, but have yielded modest results [4,7].

Also, findings from genome-wide association studies (GWAS) have identified several common genetic variants that are associated with breast cancer risk [21–27]. Approximately 70 single nucleotide polymorphisms (SNPs) have been identified to date [28,29] and some of these variants have been used cumulatively to estimate an individual’s probability of disease in an Asian context [30–34]. Despite the increase in the number of predictive SNPs, the performance of the risk prediction models are still suboptimal to be clinically useful in individualized prevention [34].

In this study, we aim to refine the Gail model by using effect sizes specific to our population, incorporating mammographic density and common genetic risk variants and evaluate the performance of the model in a prospective Singapore female cohort with large-scale mammography screening data available. In order to evaluate the potential improvement from genetics data, we simulated genotypes based on genotype frequency data relevant to the local population and averaged the results over the virtual genotype datasets. With the existing use of mammography and the decreasing costs of genotyping, we hope to utilize these readily accessible resources to build an individualized risk prediction model relevant to a developed Asian setting.

## Methods

### Study population

The subjects included in this study are women enrolled in the Singapore Breast Cancer Screening Programme (SBCSP), for which the study design has been described in detail previously [35]. The SBCSP was a prospective nationwide mammography screening project conducted between October 1994 and February 1997 among Singaporean women aged 50 to 64 years old. Eligible women were asked to complete a questionnaire regarding their demographic and anthropometric measures, family as well as reproductive history before being invited for a one-time mammogram examination. Out of 28,234 women, 3,974 did not have information on breast density because their mammograms were either unavailable, or the image quality of the scanned mammogram was too poor for the accurate assessment of mammographic density. Among those with measurements for density, 99 were detected with breast cancer at time of screening, hence, they were excluded from further analyses. Therefore, a total of 24,161 participants were used in this study. The Institutional Review Board at the National University of Singapore has approved this study. No informed consent was required as the data were analyzed anonymously.

### Density measurement

Medio-lateral oblique (MLO) views of both breasts were used in this study. Original film mammograms collected during the SBCSP, and jointly owned by the Saw Swee Hock School of Public Health and National Cancer Centre, were digitized between February 2012 through February 2013 using the 2905 Laser Film Digitizer (Array Corporation, Model 2905, Tokyo, Japan), with a sampling pitch of 50 micrometers and a gray-scale contrast resolution of 12 bits. Mammographic density was determined using a fully-automated thresholding method described previously in [36]. After images have been pre-processed such that only the breast area remains on the image, 15 global thresholding approaches available in ImageJ were applied to each image to separate the areas of “dense” breast tissue (“regions of interest”) from the remaining area of the breast. The Analyze command in ImageJ was then used to count and measure objects in the thresholded images (for groups of objects divided into four size categories: 5+ in the case of the former preprocessed images; 1 to 100, 101 to 1,000, and 1,001+ pixels, in the case of the latter images that underwent background subtraction and watershedding). A variety of measurements were obtained for the breast as a whole, as well as for the "objects" of dense tissue, under each thresholding method (see ref ImageJ paper). We also used the Analyze command in ImageJ, after applying the "find edges" filter in ImageJ to identify sharp changes in intensity, and binary thinning to find the centerlines of objects in the image (in place of thresholding). For each image, 1,008 measurements were obtained as output from ImageJ. Principal component analysis was applied on the 1,008 measurements.

Mammographic density measurements (percent density and absolute dense area) obtained by one trained observer using the semi-automated Cumulus software, currently the gold standard, were available for 2,035 images digitized using the same parameters in an independent Swedish study [36]. These measurements were used as a training set for model building with the principal components based on penalized estimation using the lasso (l1) penalty [16,17] for percent density and absolute dense area in two separate models. Estimates obtained from the respective models were then applied to estimate percent density and absolute dense area for all mammograms in the SBCSP dataset. The mean density of both breasts was calculated.

### Genotype simulation

Since biological samples had not been collected from the participants during the original study, genotypes for the 75 loci were simulated for each woman using her breast cancer status and genotype frequencies from the Asian populations in the Breast Cancer Association Consortium (BCAC) [28,29], while assuming that there is no interaction between SNPs, mammographic density and other established risk factors. S1 Table presents the list of SNPs that were used in the simulation, and their respective allele frequencies. Approximately 30 of the loci had been established in earlier GWAS, while the remaining were novel SNPs that have only been identified in the most recent BCAC findings [29]. A corresponding genetic risk score (GRS) was derived to represent the cumulative effect of all risk variants for a woman carrying a particular set of simulated genotypes. This is equivalent to the sum of (log Odds Ratio of SNP) x (Number of risk alleles that the individual carries for SNP) across all 75 SNPs. A total of 1,000 virtual genotype datasets were generated and GRS were calculated for each dataset and saved for the analysis with prediction models.

### Case ascertainment

Incident breast cancer and vital status of all participants as of 31 December 2011 was obtained via electronic linkage with the population-based Singapore Cancer Registry [37]. Both invasive and *in-situ* cases were included.

### Statistical analysis

The SBCSP questionnaire contained demographic data, reproductive risk factors as well as information on family history and past breast biopsy. We followed the risk factor categories from the original Gail model [3], where possible: age (50–54, 55–59, 60–64 years), age at menarche (≥14, 12–13, <12 years), age at first live birth (<20, 20–24, 25–29 or nulliparous, ≥30 years), number of first degree relatives (None, ≥1), and past breast biopsy (No, Yes), but used the corresponding coefficients from our study. Ethnicity (Chinese, Indian, Malay, Others) and body mass index (BMI) (<20, 20–23.9, 24–27.9, ≥28 kg/m^2^) were also considered in the model as they were relevant to our study population. Mammographic density was grouped into six dense area categories: ≤10, 11–20, 21–30, 31–40, 41–50, 51–60 cm^2^, while GRS was treated as a continuous variable.

Cox proportional hazards (PH) models were used to build the predictive models including breast density and common genetic variants and compute (approximately) 10-year risk for each individual. The validity of proportional hazards assumption was assessed by Schoenfeld residuals and all variables were not subject to time-varying effects across all the models. Three models (Gail variables + BMI, Gail variables + BMI + Density, Gail variables + BMI + Density + GRS) were constructed and compared in terms of their ability to accurately assess each woman’s 10-year absolute risk. We note that Cox PH models leave the baseline hazard un-estimated and therefore we approximated 10-year cumulative baseline hazard to compute the absolute risk (explained below). The first model consisted of variables from the Gail model, ethnicity and BMI; the second included breast density; the third had breast density and GRS. In Cox PH models, the probability that a woman will survive beyond a certain time point given a set of covariates x is calculated as $\text{S}\left(\text{t|x}\right)=\exp\left(-{\text{e}}^{\beta^{’}\text{x}}\int_{0}^{t}h\left(s\right)ds\right)$, where β’ was the corresponding effect size for each variable, and $\int_{0}^{t}h(s)ds$ was the cumulative baseline hazard for t years. In order to approximate the latter, we used the basehaz command in the survival R package, which implements the Nelson-Aalen estimator of the cumulative hazard for a person with specific covariate value x and reports the resulting survival probability estimate S^NA^(t | x). We computed this value for a person with $\bar{X}$, average values for all covariates in the model (the reference group was taken in the case of categorical variables), and approximated 10-year cumulative hazard as –log [S^NA^(t|$\bar{X}$)/(e^β’$\bar{x}$^)]. Since each subject’s 10-year absolute risk is the desired score, an individual’s 10-year risk (with covariate *X*) of being diagnosed within ten years is 1- S (10|x).

Improvement in the prediction performance was assessed by the changes in the AUC of the ROC for each model, as well as positive and negative predictive values at fixed 10-year absolute risk thresholds. We also computed the concordance probabilities using the Cox proportional hazard model fits as additional performance metric [38]. To account for overly-optimistic improvements in model performance when simulated genotypes are included, we used a 10-fold cross-validation in each of the 1,000 virtual datasets and averaged the ROCs over the 1,000 sets.

All statistical analyses were performed using R version 2.13.0. Statistical tests were two-sided and P<0.05 was considered statistically significant.

## Results

As of 31 December 2011, 680 women were diagnosed with either *in-situ* (n = 106) or invasive (n = 574) breast cancer. Table 1 shows the distribution of the study population by demographic, reproductive and other risk factors. The majority of the cases were 55 to 59 years old, more educated, have younger age at menarche, nulliparous or have their first child at a later age. They are also more likely to be current users of hormone replacement therapy, have a positive family history, previous breast biopsy, higher BMI and higher breast density. Based on the simulated genotypes, the corresponding GRS ranged from 2.75 to 7.01, with more cases being categorized in higher quintiles. The median follow-up time for cases and controls was 3131 and 6175 days respectively.

**Table 1:** Distribution of baseline characteristics in breast cancer patients (cases) and healthy individuals (non-cases).

Demographics	Cases (n = 680)	Non-cases (n = 23 481)	P value
Age, n (%)			
50–54	227 (33.4)	7111 (30.3)	<0.001
55–59	279 (41.0)	8651 (36.8)	
>60	174 (25.6)	7719 (32.9)	
Education level, n (%)			
No formal education	314 (46.2)	14 288 (60.8)	<0.001
Primary	157 (23.1)	4390 (18.7)	
Secondary or higher	255 (37.5)	4803 (20.5)	
Ethnicity, n (%)			
Chinese	583 (85.7)	19 962 (85.0)	0.07
Malay	36 (5.3)	1146 (4.9)	
Indian	38 (5.6)	1085 (4.6)	
Others	23 (3.4)	1288 (5.5)	
**Reproductive risk factors**			
Age at menarche, n (%)			
≥ 14	400 (58.8)	15 317 (65.2)	<0.001
12–13	241 (35.4)	7429 (31.6)	
<12	39 (5.7)	735 (3.1)	
Age at first live birth, n (%)			
< 20	80 (11.8)	4045 (17.2)	<0.001
20–24	202 (29.7)	8862 (37.7)	
25–29 or nulliparous	286 (42.1)	7984 (34.0)	
≥ 30	112 (16.5)	2590 (11.0)	
Age at menopause, n (%)			
< 50	308 (45.3)	11 064 (47.1)	<0.001
50–54	313 (46.0)	10 405 (44.3)	
≥ 55	59 (8.7)	2012 (8.6)	
Number of deliveries, n (%)			
0	88 (12.9)	1823 (7.8)	<0.001
1 or 2	192 (28.2)	5109 (21.8)	
3 or 4	264 (38.8)	9036 (38.5)	
≥ 5	136 (20.0)	7513 (32.0)	
Hormone Replacement Therapy (HRT) use, n (%)			
Non-user	539 (79.3)	20 349 (86.7)	<0.001
Ex-user	54 (7.9)	1378 (5.9)	
Current user	87 (12.8)	1754 (7.5)	
**Other risk factors**			
Number of affected 1st degree relatives with breast cancer, n (%)			
0	644 (94.7)	22 906 (97.6)	<0.001
1	34 (5.0)	566 (2.4)	
>1	2 (0.3)	9 (0.04)	
Past breast biopsy, n (%)			
No	615 (90.4)	22 266 (94.8)	<0.001
Yes	65 (9.6)	1215 (5.2)	
Body mass index, n (%)			
< 20	37 (5.4)	2567 (10.9)	<0.001
20 - < 24	241 (35.4)	8503 (36.2)	
24 - < 28	259 (38.1)	8273 (35.2)	
≥ 28	143 (21.0)	4138 (17.6)	
Mean breast percent density, n (%)			
< 10%	57 (8.4)	3751(16.0)	<0.001
10%—< 25%	365 (53.7)	13 243 (56.4)	
25%—< 50%	246 (36.2)	6232 (26.5)	
≥ 50%	12 (1.8)	255 (1.1)	
Mean breast dense area, n (%)			
<10 cm2	70 (10.3)	4723 (20.1)	<0.001
10–20	221 (32.5)	8646 (36.8)	
20–30	189 (27.8)	5299 (22.6)	
30–40	108 (15.9)	2755 (11.7)	
40–50	43 (6.3)	1225 (5.2)	
50–60	49 (7.2)	833 (3.5)	
Genetic Risk Score in quintiles, n (%)			
First (2.75–4.22)	73 (10.7)	4759 (20.3)	<0.001
Second (4.23–4.43)	98 (14.4)	4734 (20.2)	
Third (4.44–4.62)	144 (21.2)	4688 (20.0)	
Fourth (4.63–4.84)	152 (22.4)	4680 (19.9)	
Fifth (4.85–7.01)	213 (31.3)	4620 (19.7)	

We evaluated the associations of the Gail model predictors, ethnicity, BMI, mammographic density and GRS with breast cancer risk. From Table 2, women who are 60 to 64 years old seemed to be at lower risk of breast cancer compared to those aged 50 to 54. This protective effect is no longer present after adjustment for breast density. The Malays reported about a 30% lower risk of disease in all three models compared to the Chinese, but this did not reach statistical significance. The established Gail model risk factors remained statistically significant even after accounting for BMI, density and GRS. Every increase of 0.1 in GRS corresponds to a 10% higher risk of breast cancer on average. BMI, mean dense area, and percent density (S2 Table) also significantly increased one’s risk of disease by approximately 2 to 4 fold across all categories regardless of the prediction model.

**Table 2:** Association of conventional risk factors, BMI, mean breast dense area and GRS with breast cancer.

Variable	vGail + BMI (95% CI)	P value	vGail + BMI + Density (95% CI)	P value	vGail + BMI + Density + GRS (95% CI)	P value
**Age, years**						
50–54	1.00 (ref)		1.00 (ref)		1.00 (ref)	
55–59	1.07 (0.90, 1.28)	0.439	1.17 (0.98, 1.40)	0.078	1.17 (0.98, 1.40)	0.081
>60	0.81 (0.66, 0.99)	0.044	0.98 (0.80, 1.21)	0.886	0.98 (0.80, 1.20)	0.839
**Ethnicity**						
Chinese	1.00 (ref)		1.00 (ref)		1.00 (ref)	
Indian	1.16 (0.82, 1.62)	0.401	1.14 (0.81, 1.60)	0.453	1.10 (0.78, 1.54)	0.58
Malay	0.66 (0.43, 1.01)	0.055	0.70 (0.46, 1.08)	0.11	0.71 (0.46, 1.08)	0.112
Others	1.05 (0.75, 1.49)	0.767	1.06 (0.75, 1.50)	0.735	1.05 (0.74, 1.48)	0.797
**Age at menarche, years**						
≥14	1.00 (ref)		1.00 (ref)		1.00 (ref)	
12–13	1.14 (0.97,1.34)	0.118	1.13 (0.96, 1.33)	0.149	1.14 (0.97, 1.34)	0.119
<12	1.78 (1.28, 2.49)	<0.001	1.72 (1.23, 2.40)	0.001	1.72 (1.23, 2.41)	0.001
**Age at first live birth, years**						
<20	1.00 (ref)		1.00 (ref)		1.00 (ref)	
20–24	1.21 (0.93, 1.57)	0.166	1.16 (0.89, 1.51)	0.279	1.14 (0.88, 1.49)	0.324
25–29 or nulliparous	1.85 (1.43, 2.40)	<0.001	1.66 (1.28, 2.15)	<0.001	1.63 (1.26, 2.12)	<0.001
≥30	2.24 (1.67, 3.02)	<0.001	1.98 (1.47, 2.68)	<0.001	1.97 (1.46, 2.66)	<0.001
**Number of 1st degree relatives with breast cancer**						
None	1.00 (ref)		1.00 (ref)		1.00 (ref)	
At least 1	1.96 (1.40, 2.74)	<0.001	1.86 (1.32, 2.60)	<0.001	1.78 (1.27, 2.50)	<0.001
Past breast biopsy						
No	1.00 (ref)		1.00 (ref)		1.00 (ref)	
Yes	1.80 (1.39, 2.32)	<0.001	1.65 (1.27, 2.13)	<0.001	1.66 (1.28, 2.14)	<0.001
**Body mass index, kg/m** ^**2**^						
<20	1.00 (ref)		1.00 (ref)		1.00 (ref)	
20 to <24	2.03 (1.44, 2.87)	<0.001	2.16 (1.53, 3.06)	<0.001	2.19 (1.55, 3.10)	<0.001
24 to <28	2.40 (1.70, 3.39)	<0.001	2.64 (1.86, 3.74)	<0.001	2.66 (1.88, 3.77)	<0.001
28 or higher	2.88 (2.00, 4.17)	<0.001	3.32 (2.30, 4.81)	<0.001	3.37 (2.33, 4.88)	<0.001
**Mean breast dense area, cm** ^**2**^	NA	NA				
≤10			1.00 (ref)		1.00 (ref)	
11–20			1.62 (1.23, 2.12)	<0.001	1.60 (1.22, 2.10)	<0.001
21–30			2.21 (1.67, 2.94)	<0.001	2.20 (1.65, 2.92)	<0.001
31–40			2.38 (1.74, 3.25)	<0.001	2.33 (1.71, 3.20)	<0.001
41–50			2.11 (1.43, 3.13)	<0.001	2.12 (1.43, 3.14)	<0.001
51–60			3.30 (2.26, 4.82)	<0.001	3.27 (2.24, 4.76)	<0.001
**Genetic risk score**	NA	NA	NA	NA	1.10 (1.03, 1.16)	<0.001
(2.8–7.0)						

Note: vGail—Variables from the Gail model

**Fig 1:**
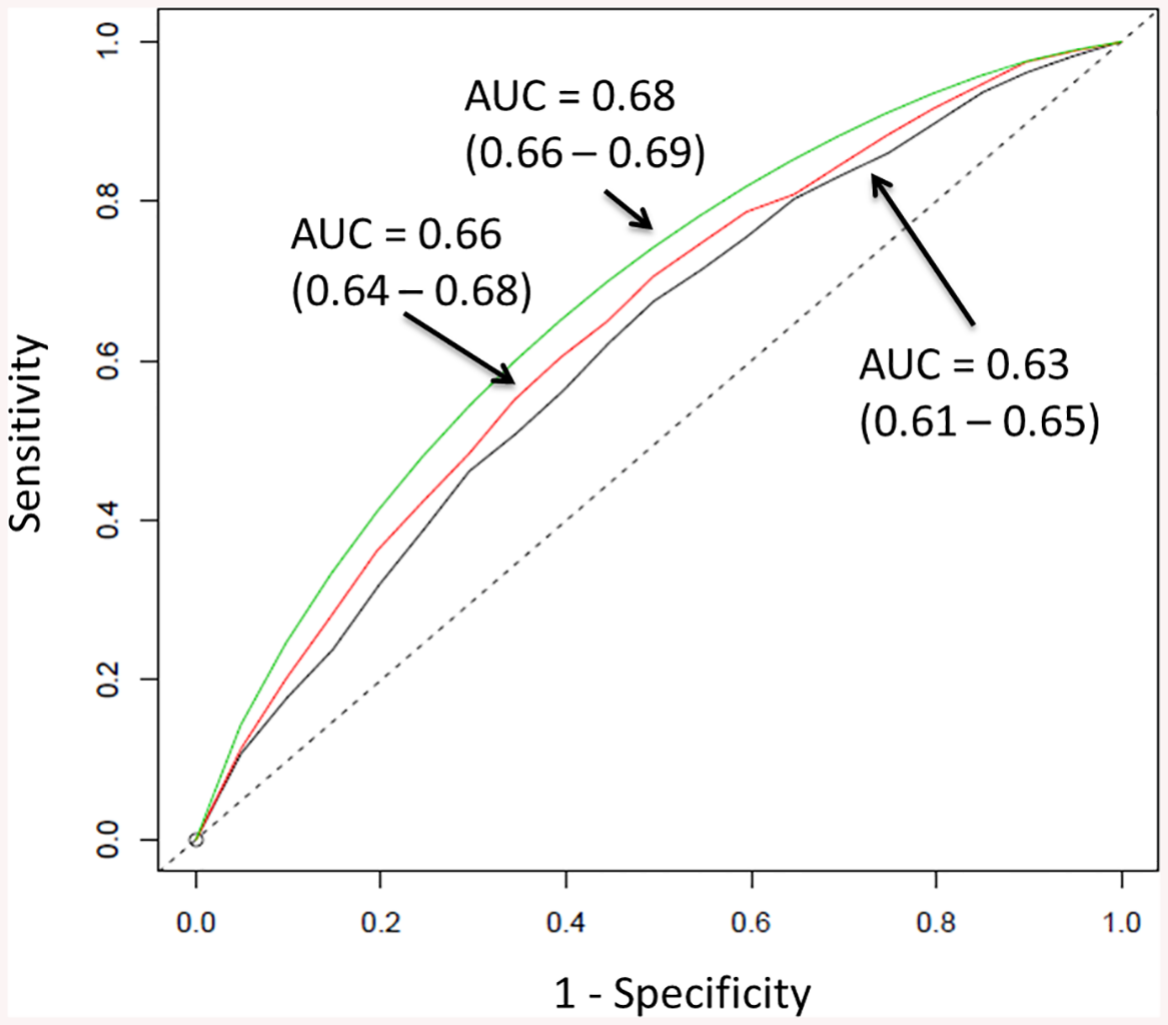
Three receiver operating characteristic (ROC) curves for predicting breast cancer: vGail + BMI (black), vGail + BMI + mean breast dense area (red), vGail + BMI + mean breast dense area + GRS (green). For the model with GRS, the average of 1000 ROC curves is drawn. Areas under the curves (AUCs) are 0.63, 0.66 and 0.68 respectively. The straight dashed line represents the ROC curve expected by chance only.

Performance in the three risk prediction models was examined by plotting ROC curves and comparing their areas under the curve. As genotypes were simulated, the average of 1,000 ROC curves for the model with GRS is reported in Fig 1. The model including Gail predictors, BMI, and mean dense area reported an area under the curve of 0.66 (0.64–0.68), while an inclusion of GRS reported 0.68 (0.66–0.69). A similar observation in model performance was observed for the same model using percent density instead (S1 Fig). Table 3 and S3 Table show the concordance probabilities for the respective models, which did not differ greatly regardless of whether mean dense area or percent density was used.

**Table 3:** Concordance probabilities of the three risk prediction models (absolute dense area).

Prediction Model	Concordance Probability	95% CI
vGail+BMI	0.62	0.60–0.64
vGail+BMI+Density	0.65	0.63–0.66
vGail+BMI+Density+GRS	0.66	0.65–0.68

**Fig 2:**
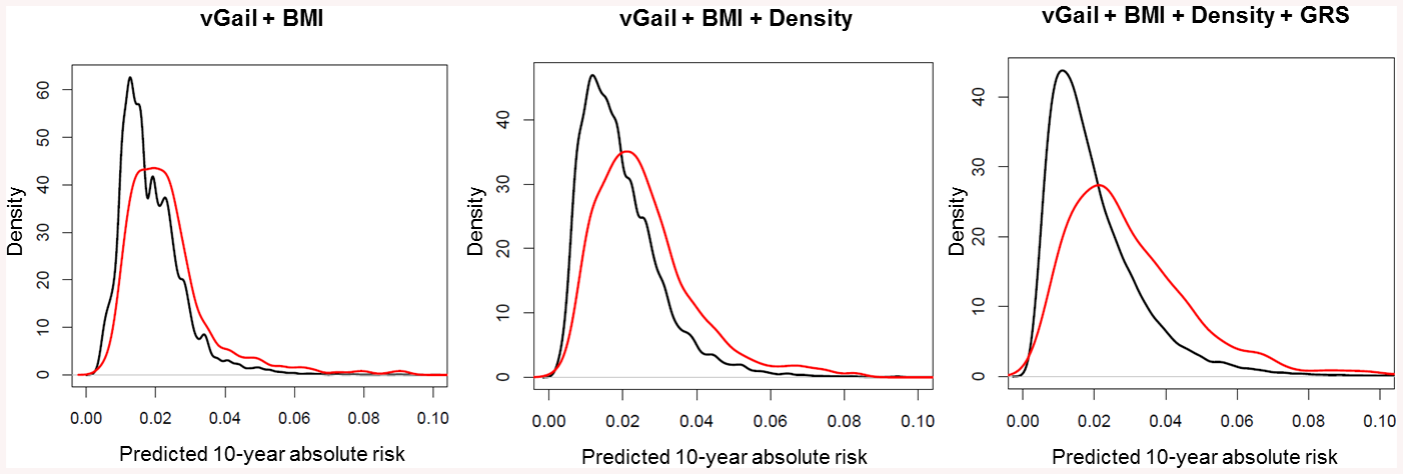
Distribution of predicted 10-year absolute risk for patients (red) and healthy individuals (black) using the three prediction models. As mean breast dense area and GRS are added to the model, the discrimination between cases and non-cases increases. Y-axis is the density which reflects the number of subjects.

From Fig 2 and S2 Fig, we note a greater discrimination between cases and controls in terms of 10-year predicted absolute risk after the addition of mammographic density and GRS. As the risk thresholds (selected *a priori*) become more stringent from 1% to 3%, the difference in the proportion of patients that are correctly identified between the modified models and the Gail model, increases in general (Table 4, S4 Table). In terms of accurately classifying healthy individuals, all models fared equally well at the first five absolute risk cut-offs, but not at 5.0% and 10.0%.

**Table 4:** Positive and negative predictive values of the three risk prediction models at five predicted 10-year absolute risk thresholds that were selected *a priori*.

Predicted 10-year absolute risk	Proportion of patients identified correctly	Proportion of healthy individuals identified correctly	Prediction model
1.0%	3.2%	98.8%	vGail+BMI
	3.3%	98.7%	vGail+BMI+Density
	3.6%	98.9%	vGail+BMI+Density+GRS
1.5%	3.9%	98.2%	vGail+BMI
	4.1%	98.4%	vGail+BMI+Density
	4.2%	98.4%	vGail+BMI+Density+GRS
2.0%	4.3%	97.9%	vGail+BMI
	4.8%	98.1%	vGail+BMI+Density
	5.0%	98.1%	vGail+BMI+Density+GRS
2.5%	4.9%	97.5%	vGail+BMI
	5.5%	97.7%	vGail+BMI+Density
	5.8%	97.9%	vGail+BMI+Density+GRS
3.0%	5.8%	97.4%	vGail+BMI
	6.1%	97.5%	vGail+BMI+Density
	6.3%	97.7%	vGail+BMI+Density+GRS
5.0%	13.3%	97.3%	vGail+BMI
	9.1%	97.3%	vGail+BMI+Density
	10.4%	97.4%	vGail+BMI+Density+GRS
10.0%	14.3%	97.2%	vGail+BMI
	20.0%	97.2%	vGail+BMI+Density
	13.6%	97.2%	vGail+BMI+Density+GRS

## Discussion

Using a prospective cohort with baseline information on risk factors, mammographic density and simulated SNPs, we have observed better prediction of 10-year cumulative risk in a low incidence environment. Models incorporating these additional predictors improved the area under the curve by 2% and 6% respectively, encouraging mammography screening and identification of more SNPs related to breast cancer risk.

The Gail model has previously been shown to be poorly calibrated to the Singapore population, over-estimating an individual’s 10-year absolute risk by close to two-fold [11]. A recent study in 2012 reported that using a revised model which was country-specific improved model performance [39]. Here, we tested the use of mammographic density in improving the performance of the established risk prediction model. Similar to past studies in other populations [4,7,40], we report a modest increase in model performance from 0.63 to 0.66, and 0.63 to 0.65 for absolute dense area and percent density, respectively (Fig 1, S1 Fig). This is slightly better than the 1% that was observed by Tice *et al*, who had assessed mammographic density with the more subjective Breast Imaging-Reporting and Data System (BI-RADS) tool, unlike the others who focused on percent density instead. Such errors in qualitative measurement tend to attenuate the association between mammographic density and risk of disease [41]. Although the best measure of mammographic density for risk assessment is still undetermined [19,42–45], the current study found no difference in AUC between the models that included either measure unless GRS was added too (Fig 1, S1 Fig). Wider discrimination between cases and controls at various 10-year absolute risk thresholds (Fig 2, S2 Fig) highlights the potential application of our modified models in a clinical setting. These thresholds were set based on the 1.7% cumulative risk of women in Singapore at age 50 [34], such that women at 3% were deemed to be at high risk. However, some of this effect could be due to the genotype data being generated independently of mammographic density and the other Gail model risk factors. The availability of a sufficient number of orthogonal predictors, each having limited predictive power, has the potential to improve the ROC curve. Given the low prevalence of breast cancer, our modified model could not enhance the existing performance of the Gail model substantially (Table 4). The proportions obtained at 5.0% and 10.0% risk thresholds may not be reliable due to the small number of individuals at these cut-offs. Given the 1.7% cumulative risk, these thresholds may not be relevant in an Asian context.

Breast cancer patients and healthy individuals were significantly different in all variables except ethnicity (Table 1). Due to the large size of the study population, the relevance of p values governing statistical significance is limited. Since the Chinese form approximately 75% of the local population, they may be over-represented in this study. Unexpectedly, ethnicity was not a statistically significant predictor in all three models as reported in earlier literature on the local population [46]. Malay women seemed to have a lower risk of breast cancer (Table 2, S2 Table) which could be explained by an overall poor response rate among those who have the disease. Alternatively, an over-diagnosis of breast cancer among the Chinese, arising from a higher utilization of mammography among this ethnic group, could have led to an ascertainment bias. The change in association of BMI with breast cancer risk, after adjustment by mammographic density, is in agreement with our understanding on its negative confounding effect [44].

To our knowledge, we are the first to consider the cumulative effect of the largest number of SNPs in breast cancer risk prediction for an Asian setting, using data from the only prospective trial conducted outside Europe and North America. Darabi *et al* have carried out a similar study recently, investigating the impact of BMI, percent mammographic density and 18 common genetic variants on Swedish post-menopausal women [40]. Their results suggest an added value in using a larger pool of genetic markers, and a more obvious shift of controls to a lower predicted risk category. Contrary to their findings, we saw a greater effect among our cases.

Most groups have initially used estimation and subsequently computer-assisted methods to measure breast density. We have applied a fully-automated, high-throughput method of measuring mammographic density, which can minimize any visual irreproducibility related to more subjective assessments. Also, this measure has been shown to be highly correlated with Cumulus, an established semi-automated tool [36].

We have made a few assumptions in our study. Firstly, since cancer notification is mandatory in Singapore and all Singaporeans have a unique identification number, we expect the completeness of reporting to be close to 100% [47]. Secondly, we assume no correlation between mammographic density and SNPs, as well as between the various SNPs. Numerous groups have found common variants that contribute to the heritability of mammographic density [48–55], but the findings have either not been replicated or been countered [56,57]. We have also adjusted for both SNPs and family history in our risk models even though their effects were likely to overlap. While the latter would also account for genetic factors that were not SNP-related, and Do *et*.*al*. had recommended the integration of both methods for enhanced accuracy [58], unnecessary or over-adjustments could lead to imprecise estimates. Thirdly, we have ignored the possible difference in screening behavior between the women in our study and those in the general population. Fourthly, when we simulated the genotypes of our subjects, we have neglected the discrepancy in genotype frequencies among the various ethnicities, as well as the linkage between certain loci that are relatively close together in the genome. Lastly, SNP ORs from published GWAS were used as HRs in our study. Even if this may not be appropriate, it was the only measure that was available.

Although we had intended to compare our modified model with the Gail model, some of our study’s categories differed from the original model. Previous breast biopsy was recorded as “Yes/No” instead of the number and presence of atypical hyperplasia; family history was coded as “None/At least 1” instead of the actual number of affected first-degree relatives. However, we believe these minor coding differences will not affect our conclusion drastically [11].

The use of SNPs is dependent on the cost of genotyping, ease of collection of genetic material through blood samples or buccal swabs, as well as the identification of additional SNPs in future. As the women from the SBCSP were recruited in the 1990s, the results from this study may not be very relevant to a cohort of individuals two decades later where lifestyle patterns are more westernized. Further studies are required to gauge the applicability of the modified models on women younger than 50 years. The modified model we propose will also not be very feasible in developing nations where resources for large-scale implementation of mammography screening and genotyping are scarce [59], or the receptivity of such procedures is low [13].

In conclusion, we have demonstrated the potential benefit of mammographic density and common genetic variants in improving the performance of an established risk prediction model in a developed Asian context. The better discriminatory power observed here may encourage future efforts to identify a large panel of novel polymorphisms and thus improve the cost-effectiveness of the current nation-wide screening program in Singapore.

## Supporting Information

S1 Fig Three receiver operating characteristic (ROC) curves for predicting breast cancer: vGail + BMI (black), vGail + BMI + mean percent breast density (red), vGail + BMI + mean percent breast density + GRS (green). For the model with GRS, the average of 1000 ROC curves is drawn. Areas under the curves (AUCs) are 0.63, 0.65 and 0.67 respectively. The straight dashed line represents the ROC curve expected by chance only.
(TIF)

S2 Fig Distribution of predicted 10-year absolute risk for patients (red) and healthy individuals (black) using the three prediction models. As mean percent breast density and GRS are added to the model, the discrimination between cases and non-cases increases. Y-axis is the density which reflects the number of subjects.
(TIF)

S1 Table List of 75 SNPs used for simulation in this study. (DOCX)

S2 Table Association of conventional risk factors, BMI, mean percent density and GRS with breast cancer. (DOCX)

S3 Table Concordance probabilities of the three risk prediction models (percent density) (DOCX)

S4 Table Positive and negative predictive values of the three risk prediction models at five predicted 10-year absolute risk thresholds that were selected a priori. Mean percent breast density is used in the prediction models.
(DOCX)
