# Supplementary figures and images for: Mammographic Breast Density and Common Genetic Variants in Breast Cancer Risk Prediction
Source: PLoS One. 2015 Sep 24;10(9):e0136650. doi: 10.1371/journal.pone.0136650 (PMC4581713; doi:10.1371/journal.pone.0136650)

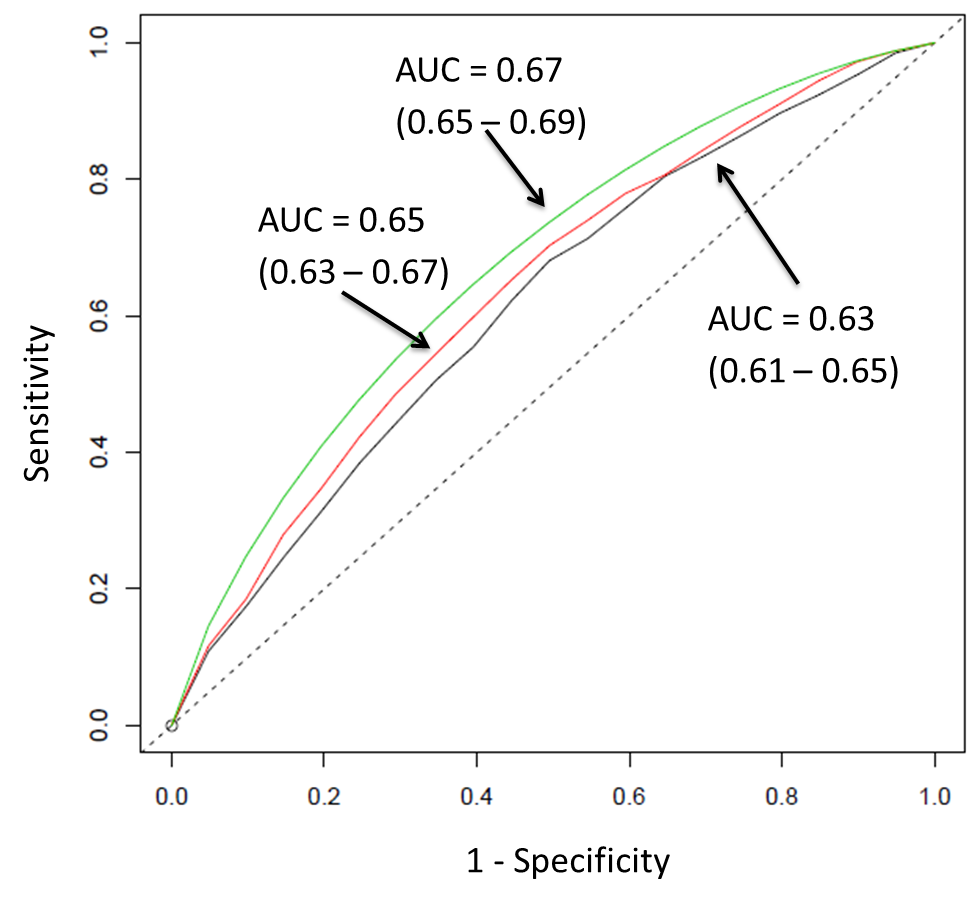

Supplement: S1 Fig — For the model with GRS, the average of 1000 ROC curves is drawn. Areas under the curves (AUCs) are 0.63, 0.65 and 0.67 respectively. The straight dashed line represents the ROC curve expected by chance only. (TIF) [file pone.0136650.s001.tif]

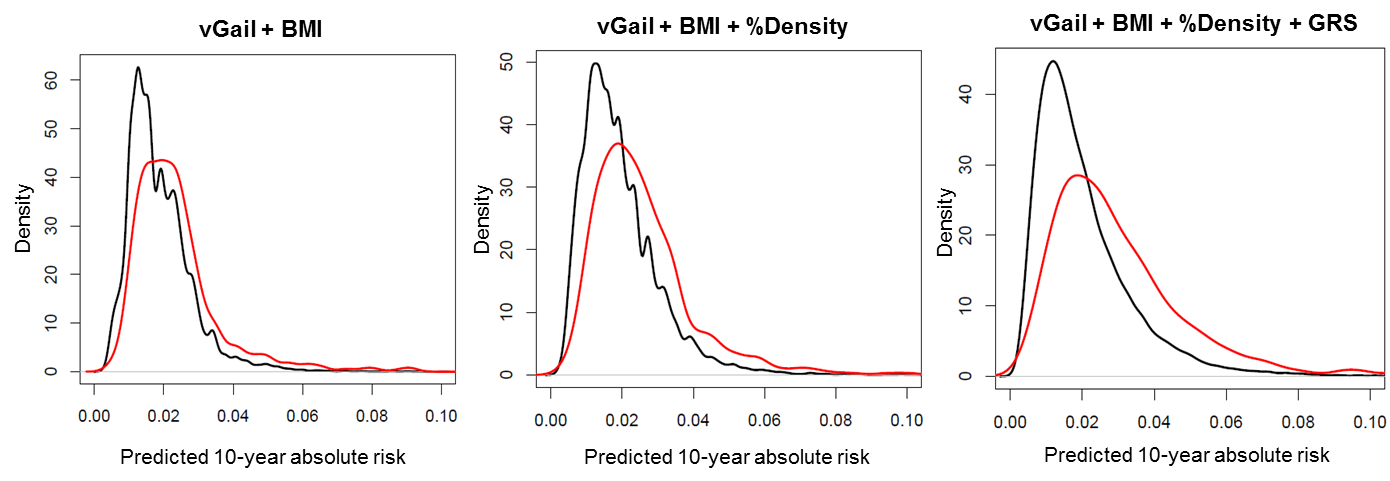

Supplement: S2 Fig — As mean percent breast density and GRS are added to the model, the discrimination between cases and non-cases increases. Y-axis is the density which reflects the number of subjects. (TIF) [file pone.0136650.s002.tif]
