# Supplementary material for: Mammographic Breast Density and Common Genetic Variants in Breast Cancer Risk Prediction
Source: PLoS One. 2015 Sep 24;10(9):e0136650. doi: 10.1371/journal.pone.0136650 (PMC4581713; doi:10.1371/journal.pone.0136650)
Supplement: S1 Table — (DOCX) [file pone.0136650.s003.docx]

**S1 Table.** **List of 75 SNPs used for simulation in this study.**

| **SNP** | **Chromosome** | **Genes in or near region** | **Position** | **Effect allele** | **Per allele OR** | **EAF among cases** | **EAF among controls** |
| --- | --- | --- | --- | --- | --- | --- | --- |
| rs12022378 | 1 | AP4B1 | 114249912 | A | 1.02 | 0.568 | 0.545 |
| rs11249433 | 1 | FCGR1B | 120982136 | G | 1.18 | 0.039 | 0.036 |
| rs616488 | 1 | PEX14 | 10488802 | G | 0.96 | 0.311 | 0.314 |
| rs4245739 | 1 | MDM4 | 202785465 | C | 0.96 | 0.050 | 0.060 |
| rs6678914 | 1 | LGR6 | 200453799 | A | 0.98 | 0.234 | 0.239 |
| rs13387042 | 2 | TNP1 | 217614077 | A | 1.05 | 0.123 | 0.125 |
| rs16857609 | 2 | DIRC3 | 218004753 | G | 0.94 | 0.379 | 0.402 |
| rs1045485 | 2 | CASP8 D302H | 201857834 | G | 1.50 | 0.001 | 0.001 |
| rs4849887 | 2 | INHBB | 120961592 | A | 0.96 | 0.206 | 0.208 |
| rs2016394 | 2 | DLX2 | 172681217 | A | 1.00 | 0.206 | 0.209 |
| rs1550623 | 2 | CDCA7 | 173921140 | G | 0.87 | 0.013 | 0.020 |
| rs12710696 | 2 | None | 19184284 | A | 1.05 | 0.330 | 0.328 |
| rs4973768 | 3 | SLC4A7 | 27391017 | A | 1.11 | 0.231 | 0.216 |
| rs6762644 | 3 | ITPR1/EGOT | 4717276 | G | 1.07 | 0.092 | 0.085 |
| rs12493607 | 3 | TGFBR2 | 30657943 | C | 0.96 | 0.327 | 0.343 |
| rs9790517 | 4 | TET2 | 106304227 | G | 1.00 | 0.400 | 0.400 |
| rs6828523 | 4 | ADAM29 | 176083001 | A | 0.94 | 0.245 | 0.245 |
| rs7726159 | 5 | None | 1335319 | A | 1.03 | 0.387 | 0.379 |
| rs10069690 | 5 | TERT | 1332790 | A | 1.04 | 0.198 | 0.197 |
| rs2736108 | 5 | TERT | 1350488 | A | 0.98 | 0.283 | 0.284 |
| rs10941679 | 5 | MRPS30 | 44742255 | G | 1.09 | 0.518 | 0.495 |
| rs889312 | 5 | MAP3K1 | 56067641 | A | 0.94 | 0.436 | 0.453 |
| rs10472076 | 5 | RAB3C | 58219818 | G | 0.99 | 0.263 | 0.270 |
| rs1353747 | 5 | PDE4D | 58373238 | C | 0.82 | 0.004 | 0.006 |
| rs1432679 | 5 | EBF1 | 158176661 | A | 0.92 | 0.351 | 0.375 |
| rs12662670 | 6 | CCDC170 | 151960549 | C | 1.18 | 0.320 | 0.274 |
| rs2046210 | 6 | ESR1 | 151990059 | A | 1.28 | 0.399 | 0.335 |
| rs11242675 | 6 | FOXQ1 | 1263878 | A | 0.93 | 0.442 | 0.465 |
| rs204247 | 6 | RANBP9 | 13830502 | A | 0.96 | 0.400 | 0.421 |
| rs17529111 | 6 | FAM46A | 82185105 | G | 1.03 | 0.196 | 0.192 |
| rs720475 | 7 | ARHGEF5/NOBOX | 143705862 | A | 0.96 | 0.038 | 0.040 |
| rs13281615 | 8 | 8q24 | 128424800 | A | 0.98 | 0.468 | 0.466 |
| rs9693444 | 8 | RPL17P33 | 29565535 | A | 1.08 | 0.309 | 0.293 |
| rs6472903 | 8 | HNF4G | 76392856 | C | 0.87 | 0.036 | 0.043 |
| rs2943559 | 8 | HNF4G | 76580492 | G | 0.94 | 0.087 | 0.096 |
| rs11780156 | 8 | MYC | 129263823 | A | 0.99 | 0.204 | 0.199 |
| rs10759243 | 9 | KLF4 | 109345936 | A | 1.06 | 0.455 | 0.438 |
| rs865686 | 9 | KLF4 | 109928299 | C | 0.94 | 0.068 | 0.075 |
| rs1011970 | 9 | CDKN2A/CDKN2B | 22052134 | A | 1.06 | 0.095 | 0.089 |
| rs2981579 | 10 | FGFR2 | 123327325 | A | 1.16 | 0.475 | 0.437 |
| rs11199914 | 10 | FGFR2 | 123083891 | A | 0.98 | 0.369 | 0.365 |
| rs7072776 | 10 | MLLT10 | 22072948 | A | 0.99 | 0.054 | 0.057 |
| rs11814448 | 10 | DNAJC1 | 22355849 | C | 1.08 | 0.011 | 0.010 |
| rs2380205 | 10 | ANKRD16 | 5926740 | A | 1.00 | 0.130 | 0.132 |
| rs10995190 | 10 | ZNF365 | 63948688 | A | 1.17 | 0.025 | 0.024 |
| rs704010 | 10 | ZMIZ1 | 80511154 | A | 1.05 | 0.340 | 0.325 |
| rs7904519 | 10 | TCF7L2 | 114763917 | G | 1.06 | 0.051 | 0.059 |
| rs554219 | 11 | None | 69040823 | G | 1.36 | 0.018 | 0.016 |
| rs3817198 | 11 | LSP1 | 1865582 | G | 1.07 | 0.143 | 0.144 |
| rs3903072 | 11 | DKFZp761E198/OVOL1/SNX32/CFL1 | 65339642 | A | 0.94 | 0.210 | 0.227 |
| rs11820646 | 11 | BARX2 | 128966381 | A | 0.96 | 0.440 | 0.440 |
| rs10771399 | 12 | PTHLH | 28046347 | G | 0.87 | 0.161 | 0.174 |
| rs1292011 | 12 | MED13L | 114320905 | G | 0.89 | 0.253 | 0.282 |
| rs12422552 | 12 | ATF7IP | 14305198 | C | 1.06 | 0.298 | 0.284 |
| rs17356907 | 12 | NTN4 | 94551890 | G | 0.92 | 0.232 | 0.250 |
| rs11571833 | 13 | BRCA2 | 31870626 | A | 2.21 | 0.001 | 0.000 |
| rs2588809 | 14 | RAD51L1 | 67730181 | A | 1.03 | 0.032 | 0.035 |
| rs999737 | 14 | RAD51L1 | 68104435 | A | 0.95 | 0.005 | 0.007 |
| rs2236007 | 14 | PAX9 | 36202520 | A | 0.93 | 0.264 | 0.275 |
| rs941764 | 14 | CCDC88C | 90910822 | G | 1.02 | 0.143 | 0.140 |
| rs3803662 | 16 | TOX3 | 51143842 | G | 0.85 | 0.369 | 0.422 |
| rs17817449 | 16 | MIR1972-2/FTO | 52370868 | C | 1.01 | 0.160 | 0.174 |
| rs13329835 | 16 | CDYL2 | 79208306 | G | 1.04 | 0.052 | 0.049 |
| rs11075995 | 16 | KIAA1752/FTO | 52412792 | T | 1.08 | 0.318 | 0.301 |
| rs6504950 | 17 | STXBP4 | 50411470 | A | 0.98 | 0.095 | 0.092 |
| rs527616 | 18 | AQP4 | 22591422 | G | 0.99 | 0.272 | 0.270 |
| rs1436904 | 18 | CHST9 | 22824665 | C | 1.00 | 0.454 | 0.439 |
| rs8170 | 19 | ANKLE1 | 17250704 | A | 1.10 | 0.004 | 0.005 |
| rs2363956 | 19 | ANKLE1 | 17255124 | C | 0.96 | 0.321 | 0.336 |
| rs4808801 | 19 | ELL | 18432141 | G | 0.97 | 0.242 | 0.256 |
| rs3760982 | 19 | KCNN4 | 48978353 | A | 0.95 | 0.179 | 0.201 |
| rs2823093 | 21 | NRIP1 | 15442703 | A | 0.91 | 0.039 | 0.043 |
| rs17879961 | 22 | CHEK2 | 27451087 | G | NA | 0.000 | 0.000 |
| rs132390 | 22 | EMID1 | 27951477 | G | 1.08 | 0.002 | 0.002 |
| rs6001930 | 22 | MKL1 | 39206180 | G | 1.05 | 0.247 | 0.240 |
