# Supplementary material for: Mammographic Breast Density and Common Genetic Variants in Breast Cancer Risk Prediction
Source: PLoS One. 2015 Sep 24;10(9):e0136650. doi: 10.1371/journal.pone.0136650 (PMC4581713; doi:10.1371/journal.pone.0136650)
Supplement: S2 Table — (DOCX) [file pone.0136650.s004.docx]

**S2 Table. Association of conventional risk factors, BMI, mean percent density and GRS with breast cancer**

| **Variable** | **vGail + BMI (95% CI)** | **P value** | **vGail + BMI + Density (95% CI)** | **P value** | **vGail + BMI + Density + GRS (95% CI)** | **P value** |
| --- | --- | --- | --- | --- | --- | --- |
| **Age, years** |  |  |  |  |  |  |
| 50 – 54 | 1.00 (ref) |  | 1.00 (ref) |  | 1.00 (ref) |  |
| 55 – 59 | 1.07 (0.90, 1.28) | 0.439 | 1.17 (0.98, 1.40) | 0.086 | 1.17 (0.98, 1.39) | 0.092 |
| >60 | 0.81 (0.66, 0.99) | 0.044 | 0.98 (0.79, 1.20) | 0.795 | 0.97 (0.78, 1.19) | 0.747 |
| **Ethnicity** |  |  |  |  |  |  |
| Chinese | 1.00 (ref) |  | 1.00 (ref) |  | 1.00 (ref) |  |
| Indian | 1.16 (0.82, 1.62) | 0.401 | 1.19 (0.85, 1.67) | 0.314 | 1.15 (0.82, 1.61) | 0.416 |
| Malay | 0.66 (0.43, 1.01) | 0.055 | 0.73 (0.48, 1.12) | 0.15 | 0.73 (0.48, 1.12) | 0.15 |
| Others | 1.05 (0.75, 1.49) | 0.767 | 1.10 (0.78, 1.56) | 0.586 | 1.08 (0.77, 1.53) | 0.646 |
| **Age at menarche, years** |  |  |  |  |  |  |
| ≥14 | 1.00 (ref) |  | 1.00 (ref) |  | 1.00 (ref) |  |
| 12 – 13 | 1.14 (0.97,1.34) | 0.118 | 1.13 (0.96, 1.33) | 0.137 | 1.14 (0.97, 1.35) | 0.105 |
| <12 | 1.78 (1.28, 2.49) | <0.001 | 1.74 (1.24, 2.43) | 0.001 | 1.75 (1.25, 2.44) | 0.001 |
| **Age at first live birth, years** |  |  |  |  |  |  |
| <20 | 1.00 (ref) |  | 1.00 (ref) |  | 1.00 (ref) |  |
| 20 – 24 | 1.21 (0.93, 1.57) | 0.166 | 1.17 (0.89, 1.52) | 0.255 | 1.15 (0.88, 1.50) | 0.294 |
| 25 – 29 or nulliparous | 1.85 (1.43, 2.40) | <0.001 | 1.68 (1.29, 2.18) | <0.001 | 1.65 (1.27, 2.14) | <0.001 |
| ≥30 | 2.24 (1.67, 3.02) | <0.001 | 2.02 (1.50, 2.73) | <0.001 | 2.01 (1.49, 2.71) | <0.001 |
| **Number of 1st degree relatives with breast cancer** |  |  |  |  |  |  |
| None | 1.00 (ref) |  | 1.00 (ref) |  | 1.00 (ref) |  |
| At least 1 | 1.96 (1.40, 2.74) | <0.001 | 1.88 (1.34, 2.63) | <0.001 | 1.80 (1.28, 2.52) | <0.001 |
| Past breast biopsy |  |  |  |  |  |  |
| No | 1.00 (ref) |  | 1.00 (ref) |  | 1.00 (ref) |  |
| Yes | 1.80 (1.39, 2.32) | <0.001 | 1.67 (1.29, 2.16) | <0.001 | 1.67 (1.29, 2.16) | <0.001 |
| Body mass index, kg/m2 |  |  |  |  |  |  |
| <20 | 1.00 (ref) |  | 1.00 (ref) |  | 1.00 (ref) |  |
| 20 to <24 | 2.03 (1.44, 2.87) | <0.001 | 2.25 (1.59, 3.18) | <0.001 | 2.27 (1.60, 3.22) | <0.001 |
| 24 to <28 | 2.40 (1.70, 3.39) | <0.001 | 2.84 (2.00, 4.03) | <0.001 | 2.86 (2.02, 4.07) | <0.001 |
| 28 or higher | 2.88 (2.00, 4.17) | <0.001 | 3.74 (2.57, 5.45) | <0.001 | 3.78 (2.60, 5.51) | <0.001 |
| **Mean breast percent density, %** | NA | NA |  |  |  |  |
| <10 |  |  | 1.00 (ref) |  | 1.00 (ref) |  |
| 10 - <25 |  |  | 1.78 (1.34, 2.37) | <0.001 | 1.77 (1.33, 2.36) | <0.001 |
| 25 - <50 |  |  | 2.59 (1.90, 3.54) | <0.001 | 2.56 (1.88, 3.49) | <0.001 |
| ≥50 |  |  | 3.20 (1.69, 6.07) | <0.001 | 3.31 (1.75, 6.27) | <0.001 |
| **Genetic risk score** | NA | NA | NA | NA | 1.10 (1.03, 1.16) | <0.001 |
| (2.8 – 7.0) |  |  |  |  |  |  |

Note: vGail - Variables from the Gail model
