# Supplementary material for: Mammographic Breast Density and Common Genetic Variants in Breast Cancer Risk Prediction
Source: PLoS One. 2015 Sep 24;10(9):e0136650. doi: 10.1371/journal.pone.0136650 (PMC4581713; doi:10.1371/journal.pone.0136650)
Supplement: S3 Table — (DOCX) [file pone.0136650.s005.docx]

**S3 Table. Concordance probabilities of the three risk prediction models (percent density)**

| **Prediction Model** | **Concordance Probability** | **95% CI** |
| --- | --- | --- |
| vGail+BMI | 0.62 | 0.60 - 0.64 |
| vGail+BMI+%Density | 0.64 | 0.62 - 0.66 |
| vGail+BMI+%Density+GRS | 0.66 | 0.65 - 0.68 |
