# Supplementary material for: Mammographic Breast Density and Common Genetic Variants in Breast Cancer Risk Prediction
Source: PLoS One. 2015 Sep 24;10(9):e0136650. doi: 10.1371/journal.pone.0136650 (PMC4581713; doi:10.1371/journal.pone.0136650)
Supplement: S4 Table — Mean percent breast density is used in the prediction models. (DOCX) [file pone.0136650.s006.docx]

**S4 Table. Positive and negative predictive values of the three risk prediction models at five predicted 10-year absolute risk thresholds that were selected *a priori.* Mean percent breast density is used in the prediction models.**

| **Predicted 10-year absolute risk** | **Proportion of patients identified correctly** | **Proportion of healthy individuals identified correctly** | **Prediction model** |
| --- | --- | --- | --- |
| 1.0% | 3.2% | 98.8% | vGail+BMI |
|  | 3.3% | 98.9% | vGail+BMI+%Density |
|  | 3.6% | 98.9% | vGail+BMI+%Density+GRS |
| 1.5% | 3.8% | 98.2% | vGail+BMI |
|  | 4.1% | 98.4% | vGail+BMI+%Density |
|  | 4.3% | 98.4% | vGail+BMI+%Density+GRS |
| 2.0% | 4.3% | 97.9% | vGail+BMI |
|  | 4.7% | 98.0% | vGail+BMI+%Density |
|  | 5.0% | 98.1% | vGail+BMI+%Density+GRS |
| 2.5% | 4.9% | 97.5% | vGail+BMI |
|  | 5.3% | 97.7% | vGail+BMI+%Density |
|  | 5.8% | 97.9% | vGail+BMI+%Density+GRS |
| 3.0% | 5.8% | 97.4% | vGail+BMI |
|  | 5.9% | 97.5% | vGail+BMI+%Density |
|  | 6.4% | 97.7% | vGail+BMI+%Density+GRS |
| 5.0% | 13.3% | 97.3% | vGail+BMI |
|  | 10.4% | 97.3% | vGail+BMI+%Density |
|  | 10.7% | 97.4% | vGail+BMI+%Density+GRS |
| 10.0% | 14.3% | 97.2% | vGail+BMI |
|  | 28.6% | 97.2% | vGail+BMI+%Density |
|  | 16.3% | 97.2% | vGail+BMI+%Density+GRS |
